# Supplementary material for: Integrating perspectives of patients, healthcare professionals, system developers and academics in the co-design of a digital information tool
Source: PLoS One. 2021 Jul 16;16(7):e0253448. doi: 10.1371/journal.pone.0253448 (PMC8284604; doi:10.1371/journal.pone.0253448)
Supplement: Attachment — Submitted filename: Respons to Reviewers.docx [file pone.0253448.s001.docx]

|  | **Journal Requirements:** | **Response:** |
| --- | --- | --- |
|  | Please ensure that your manuscript meets PLOS ONE's style requirements, including those for file naming | - Revised - Corrected according to formatting Guidelines |
|  | Please amend your Methods section to include all of the information about ethics approval and consent that you provided in the Ethics Statement. | Revised |
|  | Please amend either the title on the online submission form (via Edit Submission) or the title in the manuscript so that they are identical. | Revised |

|  | **Reviewer 1 Comments:** | **Response:** | **Revised text in manuscript:** |
| --- | --- | --- | --- |
| 1 | **Abstract**: Methods, results and discussion are missing. The abstract ends with the study question which should rather be at the beginning. | Thank you for this important suggestion. The abstract has been revised according to this. Please see amended text in the next column. | ***Abstract***  ***Background***  *Patients diagnosed with cancer who are due to commence radiotherapy, often, despite the provision of a considerable amount of information, report a range of unmet information needs about the treatment process. Factors such as inadequate provision of information, or the stressful situation of having to deal with information about unfamiliar things, may influence the patient’s ability to comprehend the information. There is a need to further advance the format in which such information is presented. The composition of information should be tailored according to the patient’s individual needs and style of learning.*  ***Method and findings***  *A description of the development, applying participatory design methodology, of a new digital information tool*  *In co-design with patients, healthcare professionals, system developers and academics participated as stakeholders we have developed a digital information tool. The digital tool is based on Virtual Reality technology to in an innovative, graphical way prepare patients affected by cancer for radiotherapy treatment. Involving different stakeholders in co-designing a digital information tool proved to be valuable for the research process and development of the tool.*  ***Conclusions***  *Involving the end-users, the co-design of developing information material, proved to be beneficial and enhanced the rigor of the process, and ensured the relevance of the information, furthermore with potential to employ a positive effect on the reach of the related material.* |
| 2a | **Introduction**: (a) The primary goal of the intervention is not clear as information, increased perception, health literacy and informed decisions are surely different aims. | - Thank you for noting this. We have made amendments to the background to enable the reader to easily follow that although we apply PD it is nothing that we will measure. This is original research that is more descriptive of a kind hence the method and results are mixed. Please see revised text in the next column. (Line 148 in “Manuscript” document.) - To measure or explore information, increased perception, health literacy and informed decision is not aim of this particular research article. This article is part of a larger study. A Study protocol manuscript for the larger study including an RCT is currently under review (see below). This article is more of a foundation/ groundwork where we are describing the process of developing a digital information tool applying co-design. For clarification, the different variables including health literacy, distress, will be measured within the larger prospective RCT study. The results will be presented in future research articles. - Within the scope of this article, we will describe the method according to PD. However, the concepts mentioned are used as a theoretical background to the whole project, where the development of the digital tool is one part. | *It is worth to note that although PD is applied it is nothing that we will measure within the scope of this article. This is original research that is more descriptive of a kind.* |
| 2b  2b | (b) The aim of the paper is given as “to describe the PD process”. This is not sufficient, as this does rather imply a study protocol than a scientific study. | - Although being original research, this is not a traditional scientific study with an intervention which aim to test something. Similar to Smith F, Wallengren C, Öhlén J. Participatory design in education materials in a health care context. Action Research. 2017;15(3):310-36, we aim to describe a PD process applying co-design. - Further articles describing this are Clemensen et al. (2017). Participatory design methods in telemedicine research. Journal of Telemedicine and Telecare. - We have now, according to your suggestion, amended (line 143) to clarify that this article is part of a larger RCT. Please see revised text in the next column. - As mentioned above in this article we describe the process of developing a digital information tool applying co-design. It is, however, of importance to also present the processes of co-design of an artefact since it is part of a greater study and co-design per se is a valuable research area. - There is a Study protocol manuscript that currently are under review Fristedt, F., Smith, F., Grynne, A., Browall, M. (in manuscript). Digi-Do – a digital information tool to support patients with breast cancer before, during, and after start of radiotherapy treatment: an RCT study protocol (line 249). | *This article is part of a larger prospective Randomized Control Trial (RCT) study and constitutes of a foundation and groundwork for the project.*  *The information gained from reading the existing patient information together with the results from the workshop and patient survey, constituted the protocol document, which also describes the design of the project, the rationale, and the process for the development of the digital information tool [34].* |
| 3a | **Methods**: (a) Has the study protocol of the main study been published or registered? Please provide references, if applicable. | There is a Study protocol manuscript that is currently under review (as above). |  |
| 3b | **Methods**: (b) The chosen participatory design seems adequate, but there need to be more details especially concerning recruitment of participants (how, when, where from…) and rationale for sample sizes. It might be the case that all these have been pragmatic/convenience processes, but this should be stated. | - Thank you for highlighting this. The text under methods have been revised and includes a heading “Setting, participants and procedure”. We have also made amendments to the method section to include a short method description of each step in the process. (Line 206). - One of the researchers in the project management group had a role as clinical associate professor at the RT department. Together with the manager for the RT department she was able to do a strategic selection of staff to ensure a broad sample of professionals participating in the different steps. | ***Initial exploration of work***  *Step 1 - One of the researchers in the project management group (KA) had a role as clinical associate professor at the RT department. Together with the manager at the RT department KA was able to do a strategic selection among the staff to ensure a broad sample of professionals participating in the focus group interview. The staff was informed about the research and following verbal consent invited to participate in the workshop for an initial assessment. In the workshop they described what they, in their specific role, experienced were essential to know from a patient’s perspective due to commence RT treatment. Through a strategic selection the researcher (KA) invited patients undergoing RT treatment to participate in a patient survey. Additionally, in this step existing written patient information (booklets) were identified and examined. The project management team wanted a strategic selection of individuals who could contribute to the co-design and development of the digital information tool. It was important that healthcare professionals, administration staff, IDT staff and patients, all voices to be heard in accordance with PD methodology.*  ***Discovery process***  *Step 2 – The IDT with extensive experience in health information innovations was contracted to produce the digital tool version 1.0.*  *Step 3 – The relevance, comprehensiveness, feasibility, and usefulness of the digital tool version 1.0 was tested and assessed in a pilot study.*  ***Prototyping***  *Step 4 - Two focus groups were conducted with health care professionals and staff from the innovation development team. A semi-structured interview with predesigned questions was used at the focus groups. The participants were asked to share their experiences of how they considered their experience working with researchers to develop a digital tool and how they experienced the patients following using the digital tool. A thematic analysis was applied.* |
| 3c | **Methods**: c) The “Elwyn protocol” is given as one of the underlying approaches. First, I wonder if the term “Elwyn protocol” is appropriate, but more important, the framework cannot clearly be found here and as it addresses decision support interventions, does not clearly fit the proposed intervention here. | - Constructing patient information is not an easy task. To be transparent about the work process we applied a protocol developed by Elwyn. This was to clarify all steps within the process. The protocol has been modified to better suit the indented project. Linden et al (2012) present similar set up in their article. The text has been revised (line 184). - The protocol developed by Elwyn has previously been used by the research group. | *..was, in relation to this article, combined with a protocol developed by Elwyn [33],* |
| 3d | **Methods**: (d) As stated above, under “Work Procedure”, important information about recruitment procedures are missing. Also, there are aspects in the results section e.g., on the conduction of the group interviews that rather belong here. For most parts of the study, methods are not reported (in detail), e.g., on the methods used for conducting the interviews and the qualitative analysis methods. Also, for the literature search and the “evidence synthesis”, methods are not reported. The procedure for identifying and analyzing “existing written information” also remains unclear. Also, the “pilot project” remains unclear as this seems to have been a controlled study, but there is no further information on this. The reported results therefore cannot be interpreted. | - This has been revised in the text according to your suggestions. - Semi-structured interview guide with predesigned questions was used in the focus group interviews. (Line 226). - We apologies if we gave the impression of using qualitative content analysis methods, this was not the case, thematic analyze was applied. In our RCT study we will conduct interviews where we plan to analyze the text with content analysis. - Written information – we apologize if this is not very clear and thank you for pointing this out. The written information was developed by healthcare professionals at the RT department who through evidence synthesis applying evidence-based information when creating booklets for patients with focus on cancer and RT. This has now been corrected. (Line 245). - The existing written information was read through, examined, and transferred into the digital information tool, the information app in form of “questions and answers”. Also, additional findings from the workshop, interviews with patients were adopted into information to suit the information app. - Within the protocol developed by Elwyn et al the concept “evidence synthesis” is applied. - In this article the pilot study was undertaken to assess the relevance, comprehensiveness, feasibility, and usefulness of the digital tool. - The results from the control group were not used in this article more for the design for the upcoming RCT. We only present feasibility test. For clarification this has been removed from the manuscript. | - *Please see above box 3b.* - *Step 4 - Two focus groups were conducted with health care professionals and staff from the innovation development team. A semi-structured interview with predesigned questions was used at the focus groups.* - *Finally, existing written patient information material consisting of evidence-based information in form of booklets with focus on cancer and RT used at the hospital was identified and examined. The existing written patient information material was developed by healthcare professionals at the RT department following an evidence synthesis of the literature.* |
| 4 | **Results**: This section contains a lot of methods content (see above), which also highlights the lack of presented results. | This is correct; however, the design of this study is of descriptive origin hence there is no result in form of traditional research articles as such. In order to provide a better flow of text, the methods and results are presented together. This has been clarified in the text of results section. Please see revised text in the next column. | *The design of this study is of descriptive origin and to enable a better flow of the text, the methods and results are presented unitedly. The flow of the structure of the different steps are illustrated in Figure 1*. |
| 5a | **Discussion**: (a) I acknowledge that the study aimed for a “transparent description of the co-design process…”, which is valuable, but can at present not be derived from the paper. Also, there are many aspects that seem to belong to the background or are repetitions (e.g., “Engaging different stakeholders in the research process can improve the rigor, relevance, and reach of science”) (l.320-321), which was clear from the outset.  The same applies to several other paragraphs, e.g., just to state another one “However, although adopting to a co-design process may be time-consuming, the consequences of not involving stakeholders and not testing the digital information tool in a pilot project must be considered, as this will have a detrimental effect on the quality and usability of the end product.” This is nothing that was shown by this study. | - The co-design process has been described within the scope of the article described in step 1. - In the result section it is emphasized that results from this process confirms the importance of involving the end-users and this is being supported by Balazs & Morello-Frosch (2013) and the important relation between PD and the three Rs. - Going through the different steps enabled us to gain more of an understanding of what information is relevant and important for the end-users, information that relates to other subjects than medical knowledge. - This is now corrected to “*may* have a determinate effect” - we have not tested the digital instrument in this study – we are currently doing this in an ongoing RCT. We are here backing up this sentence with previous literature (Smith et al 2017). (Line 395). - The project commenced in 2017, and the pilot study was undertaken spring 2018. The process developing the digital tool was lengthy; version 1 to current version 2. In the focus group interview with staff from IUD they pointed out that the fast pace of advancement of the technology is a challenge in relation to the, at times, slow research process. A section has been added under Method with heading “Setting, participants and procedure” to better present this. Please see next column. (Line 171). - Furthermore, involving digital technology in the research process may impose challenges unable to foresee from the start for example in our present RCT the technical circumstances, out of our control, changed as Apple made a change to iPhone in their latest update version which had the implication that we were no longer able to use “navigation with VR-glasses” of the digital information tool with iPhone. | - *However, although adopting to a co-design process may be time-consuming, the consequences of not involving stakeholders and not testing the digital information tool in a pilot study must be considered, as this may have a negative impact on the quality and usability of the end-product [8].* - *This project took place in a RT-department in a Universitas hospital in Sweden. The project described within this article commenced 2017, and the pilot study was undertaken spring 2018. The design of this study is of descriptive origin and to enable a better flow of the text, some of the methods and results are sometimes presented unitedly.* |
| 5b | **Discussion**: (b) In line 345 again the “positive effects” are mentioned, and I am not sure what this refers to. | Thank you for pointing this out. The text has now been revised and its hopefully made clearer what the “positive effects” relates to. (Line 367). | *The approach also included the families involved, as the patient effortlessly was able to share the digital tool with others. Some patients in the pilot study experienced a greater understanding and support from the family as they by using the digital tool gained more of an insight of learned more about living with cancer and RT.* |
| 5c | **Discussion**: A section describing strength and limitation of this study is missing. For example, the fact that no patients participated in the initial workshops should be stated in this context as well as the (proposedly) convenience sampling. In contrast, only limitations of the developed interventions are stated, which is interesting, but of minor relevance for this paper. | Thank you for highlighting this. This has now been corrected. (Line 464). Please see revised text in the next column. | ***Strengths and weaknesses***  *A strength of this article is the transparent description of the PD process presenting the involvement of patients, healthcare professionals and the IUD team as co-designers which ensured the three R’s in the project. While something that can be viewed as a limitation is that the research team was unsuccessful in recruiting patients to the initial workshops. Even though the patient participants need of information was measured through a patient survey and complementary individual short briefings their lack of participation in the initial workshops may have resulted in missing valuable information regarding the patient’s perspective of information needs related to RT treatment. What also may be seen as a limitation and risk for introducing bias is that the patient representative was a member of the IDT. By being aware of this was addressed and discussed during the process.* |
| 6 | **Conclusion**: Here, you even refer to “…a greatly positive effect”, please clarify and present data or revise. | You are correct in your observation and we can see what you mean. Thank you for pointing this out. Please see revised text in the next column. | *What we can conclude from our research is that involving different stakeholders who are the end-users throughout the research process, although time-consuming, is worthwhile, as this approach had a positive effect on the rigor, relevance, and reach of the project in form of development of the digital tool including relevant information material, the design and interfaces of the tool and the future implementation process.* |
| 7a | **Figure**: (a) The figure is very helpful, and I would suggest to structure both the methods and the results sections, accordingly, using the same headlines as used in the figure. | We have made revised the headings and the structure with headlines are now applied in both methods and results sections to present the different steps of the process. |  |
| 7b | **Figure**: (b) Typo: RTC. | Thank you for pointing this out. This has now been amended. |  |

|  | **Reviewer 2 Comments:** | **Response:** | **Revised text in manuscript:** |
| --- | --- | --- | --- |
| 1 | It is unclear why the authors chose Spinuzzi as reference for co-design methodology, which is a bit old (2005) while the majority of patient engagement methodologies and best practices have been developed in the last five years. | You are right that there has been some great development within patient engagement methodologist. However, Spinuzzi (2005) is still valid and clearly describes the theory and contribution of PD. We also believe it to be well accustomed to the more step by step actions described in the protocol developed by Elwyn et al. Hence, we are using Spinuzzi (2005) as we in the article describe the use of PD methodology in the development of a new digital information tool. We are referring to Smith et al (2017) who writes about PD, we have also added Simonsen & Robertson (2013) as reference in the text. |  |
| 2 | No evidence that a review has been performed (even a quick one) of publications on patient involvement or co-design methodologies. No evidence of awareness of important patient engagement projects at European level like PARADIGM or PREFER, that, although focused on drug development and not innovative solution development, provide good Frameworks for patient engagement validated at multi-stakeholder level. | - We have focused on the development of patient information and web-based artifacts. Within the scope of this article, we have focused on PD. We agree that there are many important projects both on national and international level however, we have focused on specific subject of interest. - We refer to appropriate literature related to co-design Please see few examples in the next column of articles being referred to in the article. - Thank you for the advice and interesting recommendations, we will look into these engagement project for our future projects and when presenting the results from the RCT study. | - *Smith F, Wallengren C, Öhlén J. Participatory design in education materials in a health care context. Action Research. 2017;15(3):310-36. Batalden M, Batalden P, Margolis P, Seid M, Armstrong G, Opipari-Arrigan L, et al. Coproduction of healthcare service. BMJ Quality & Safety. 2016;25(7):509. Clemensen et al. Participtory design methods in telemedicine research. Journal of Telemedicine and Telecare. 2017;23(9): 780-785.* |
| 3 | Governance: in the Project Management group no patients have been included, just researchers. Patient Experts should have been involved.  The patient rep in the IDT group was also an employee, which could introduce bias. | - We appreciate that you voice a concern regarding the patient’s involvement, and we agree that their involvement is imperative. However, in the protocol applied to the work of this article, developed by Elwyn it is clearly stated the role of the project management group and the role of the advisory group. The management group is the group that has typically obtained the resources for the project, written the protocol document and specification document for the design of the project. It is a role that involves engagement in a different to the role as a stakeholder in an advisory group. - We have work according to the PD process and throughout the process and within this article we have been transparent regarding the patient’s involvement working according to a set protocol and where we have seen the need for further involvement. - Within the scope of this project, we aim to describe the development of a digital information tool applying co-design using PD methodology. We have therefore involved patients in the initial assessment needs, we have also tested the feasibility of the tool in the pilot study. As much as we appreciate and agree that your suggestion to involve patients in the project management group would be valuable, we decided to involve the patients in the initial assessment and test of feasibility of the tool in the pilot study only due to the fact stated in the protocol developed by Elwyn. - We agree that it may be a risk to introduce bias using the patient represent in the IDT team, but her professional role was from the beginning clear as she was mostly involved as the speaker for the voice-over. This however was discussed continuously, and person felt she was able to keep the two roles separate. This has been added as a limitation. | *What also may be seen as a limitation and risk for introducing bias is that the patient representative was a member of the IDT. By being aware of this was addressed and discussed during the process.* |
| 4 | Patients have not been involved in the review of existing written information. | - This is a correct observation, and we appreciate the text is not very clear. The written information is developed by healthcare professionals applying evidence-based information in the patient information with focus on cancer and RT. This has now been corrected. Thereby no further review of the existing written information took place, but the existing literature was read through, examined, and transferred into the information app of the digital information tool, in form of “questions and answers”. (Line 245). - In step 1, in this article, patients were asked in the survey how they experienced the provided written information. - Within the scope of this article, we aim at describing the co-design process where the patients have been involved in the initial design of the tool, they have been involved in testing the feasibility of the tool (pilot study), including the information “questions and answers” in the information app. - The information of the information app in the digital information instrument will be assessed and evaluated in the prospective RCT study. | *Finally, existing written patient information material consisting of evidence-based information in form of booklets with focus on cancer and RT used at the hospital was identified and examined. The existing written patient information material was developed by healthcare professionals at the RT department following an evidence synthesis of the literature.* |
