# Supplementary material for: Integrating perspectives of patients, healthcare professionals, system developers and academics in the co-design of a digital information tool
Source: PLoS One. 2021 Jul 16;16(7):e0253448. doi: 10.1371/journal.pone.0253448 (PMC8284604; doi:10.1371/journal.pone.0253448)
Supplement: Attachment — Submitted filename: Respons to Reviewers.docx [file pone.0253448.s002.docx]

|  | **Journal Requirements:** | **Response:** |
| --- | --- | --- |
|  | Please review your reference list to ensure that it is complete and correct. | Reference Fristedt et al 2021 has been successfully accepted and published. The reference list has been updated accordingly. |
|  |  |  |
|  |  |  |

|  | **Reviewer 1 Comments:** | **Response:** | **Revised text in manuscript:** |
| --- | --- | --- | --- |
| 1 | General: The wording in the newly added passages sometimes seems not completely adequate, but being not a native speaker myself, I would leave this to the copy editors. | Thank you for highlighting this. The text has been checked again and hopefully reads better now. |  |
| 2 | Abstract: In my view, the abstract is still not acceptable. To combine methods & results is neither adequate nor necessary. In addition, there is hardly any information about the PD and its results and implications. Instead, there is information about the digital tool (which is not the focus of the article) and some redundancy (e.g. “Involving…proved to be valuable” & “Involving…proved to be beneficial”). Please revise and clearly state study results. | You have got a valuable point; I have now amended the text in the abstract to include the PD more specifically. Please see revised text.  I very much appreciate your view; however, I still would like to argue that this is original research that is more descriptive of a kind hence the method and findings are mixed. | **Method and findings**  The PD methodology is frequently used when a technology designed artefact is the desired result of the process. This research is descriptive of its kind and provides a transparent description of the co-design process used to develop an innovative digital information tool employing PD methodology where several stakeholders participated as co-designers. Involving different stakeholders in the process in line with recommended PD activities enabled us to develop a digital information tool that has the potential to be relevant and user-friendly for the ultimate consumer.  **Conclusions**  Facilitating collaboration, structured PD activities can help researchers, healthcare professionals and patients to co-design patient information that meets the end users’ needs. Furthermore, it can enhance the rigor of the process, ensure the relevance of the information, and finally have a potential to employ a positive effect on the reach of the related digital information tool. |
| 3 | L.167: “PD is about the direct involement (sic!) of people in the co-design…” As co-design is about involving people, I think, this sentence does not make sense. | You are of course correct about this. I have now amended the text. | L 157: PD is an approach that seek to actively involve all stakeholders in the design process and is often used when a technology designed artefact is the desired result of the process. |
| 4 | L.385-7: At some instances, the authors tend to describe the result of the process rather than the process, e.g. “Our article is one of the first to present a digital information tool that includes VR-technology.” As the paper is not about the tool, but the process, this seems incorrect, please check. | Absolutely, I agree with you that the article is about “the process”. I have removed the first sentence in the discussion accordingly and it now reads as you can see in the next column. | L 348: Within the scope of this article, we provide a transparent description of the co-design process used to develop an innovative digital information tool employing PD methodology where several stakeholders participated as co-designers. |
| 5 | L.506: “A strength of this article…” This section should target the strength of the study, not the paper, please adapt.+ | You are of course right, and I agree that is the way to write in a traditional scientific study. This however, although being original research is not a traditional scientific study, we do not aim to test something here, we aim to describe the PD approach with co-design. |  |
| 6 | L.508: The 3 R’s are mentioned (as such) for the first time here, please use this abbreviation above. | To seek to clarify for the reader, I have now amended the text where I am initially writing about the rigor, reach and relevance of science and put in the 3 R’s in brackets.  I have also amended the text according to your suggestion – thank you very much. | L. 375: Engaging different stakeholders in the research process can improve the rigor, relevance, and reach of science (the 3 R’s).  L. 467: A strength of this article is the transparent description of the PD process presenting the involvement of patients, healthcare professionals and the IUD team as co-designers which ensured the 3 R’s in the project. |
| 7 | L.542: Was there really no funding (including the RCT)? | I apologise for being unclear with regards to funding.  This article is part of my PhD. The project, development of the digital tool and RCT has been received from Regional Cancer Centre West (RCC), Chalmers innovation office, Knut and Ragnvi Jacobsson Family Foundation and Jönköping University, IMPROVE. | The project was funded by the Regional Cancer Centre West (RCC West), Sweden, the Chalmers Innovation Office, Sweden, Knut and Ragnvi Jacobsson Family Foundation, Sweden, Jönköping University, IMPROVE, Sweden. |
| 8 | Figure: I am not completely sure what led to the changes in the figure, but they seem o.k., except the term “Prospective RCT study”, please use “Randomized controlled trial”. | Ah, there are no great changes other that I after you previously pointed out my spelling mistake on RCT in your first review where I had written RTC.  Randomized controlled trial reads better than “prospective RCT study” thank you. | Randomized controlled trial. |
